# Making trials matter: pragmatic and explanatory trials and the problem of applicability

**DOI:** 10.1186/1745-6215-10-37

**Published:** 2009-06-03

**Authors:** Shaun Treweek, Merrick Zwarenstein

**Affiliations:** 1 Division of Clinical and Population Sciences and Education, University of Dundee, Dundee, UK; 2 Norwegian Knowledge Centre for the Health Services, Oslo, Norway; 3 Centre for Health Services Sciences, Sunnybrook Research Institute, Sunnybrook Health Sciences Centre, Toronto, Ontario, Canada; 4 Institute for Clinical Evaluative Sciences, Toronto, Ontario, Canada; 5 Department of Health Policy, Management and Evaluation, University of Toronto, Toronto, Ontario, Canada

## Abstract

Randomised controlled trials are the best research design for decisions about the effect of different interventions but randomisation does not, of itself, promote the applicability of a trial's results to situations other than the precise one in which the trial was done. While methodologists and trialists have rightly paid great attention to internal validity, much less has been given to applicability.

This narrative review is aimed at those planning to conduct trials, and those aiming to use the information in them. It is intended to help the former group make their trials more widely useful and to help the latter group make more informed decisions about the wider use of existing trials. We review the differences between the design of most randomised trials (which have an explanatory attitude) and the design of trials more able to inform decision making (which have a pragmatic attitude) and discuss approaches used to assert applicability of trial results.

If we want evidence from trials to be used in clinical practice and policy, trialists should make every effort to make their trial widely applicable, which means that more trials should be pragmatic in attitude.

## Introduction

The statistical experiment, or, as we know it in medicine, the randomised controlled trial (RCT) is among the more beautiful intersections between man and mathematics. RCTs minimise the risk of bias (threats to internal validity), particularly selection bias [1,2] and are thus the best research design for decisions about the effect of different interventions, be they treatments, therapies or delivery methods and policies. But, as Cochrane noted, there is a snag: randomisation does not, of itself, promote external validity; that is, the applicability of a trial's results to situations other than the precise one in which the trial was done [3]. It is thus possible for a trial to be free of bias but be of no relevance beyond the immediate setting, patients, and practitioners among whom it was conducted. This question of applicability is central to those who have to choose between therapies for groups of patients (policymakers), for their own patients (clinicians) or for themselves (patients and families). How likely is it, these decision makers may ask, that this treatment (apparently successful in this trial or review) will achieve important benefits in my context, administered to me by my clinicians, by me to my patients, or by clinicians to patients in my organisation? In other words, 'Are these published findings applicable to my decision?'.

This narrative review is aimed at those planning to conduct trials, and those aiming to use the information in them. It is intended to help the former group make their trials more widely useful and to help the latter group make more informed decisions about the wider use of existing trials. We review the differences between the design of most RCTs and the design of trials more able to inform decision making, discuss some approaches used to assert applicability and end by proposing:

1. That applicability should be explicitly considered by trialists as they plan their trial, and by decision makers when gathering evidence to guide decisions.

2. That trialists explicitly design their trials to produce results that are more widely applicable than is the case at present.

3. That trialists can and should report their trials in ways that make it easier for others to make judgements about their applicability.

4. That decision makers seek trials with a pragmatic attitude to inform choices that directly affect clinical care, health services delivery and health policies.

5. That researchers conduct, and research funders fund, empirical research to understand the major determinants of applicability.

## Explanatory and pragmatic attitudes to trials

Two French statisticians, Schwartz and Lellouch, were acutely aware over 40 years ago of the limited applicability of many trial results beyond the artificial, 'laboratory' environment of the trial [4]. They proposed a distinction between trials aimed at confirming a physiological hypothesis, precisely specified as a causal relationship between administration of an intervention and some physiological outcome (which they called 'explanatory') and the entirely different group of trials aimed at informing a clinical, health service or policy decision, where this decision involves the choice between two or more interventions (called 'pragmatic').

While explanatory trials have an important role in providing knowledge concerning the effects of precisely defined interventions applied to select groups under optimal conditions, healthcare interventions are seldom given under such circumstances [5,6]. Moreover, inadequate consideration of applicability is the most frequent criticism by clinicians of randomised trials, systematic reviews and guidelines [7,8]. For example, a clinician considering a treatment for secondary prevention of stroke might read results from the Heart Outcomes Prevention Evaluation (HOPE) trial and wonder what to make of the long list of exclusion criteria, the exclusion of nearly 1 in 10 of the remaining patients because of non-adherence, side-effects or withdrawal of consent in the pretrial run-in phase, and the use of placebo as comparator rather than aspirin [9]. Calls for more trials with wide applicability have come both from those interested in improved treatment for clinical problems [10-12] and those interested in health policy [13,14].

Schwartz and Lellouch characterised pragmatism as an attitude to trial design rather than a characteristic of the trial itself. Although some authors appear to suggest that a trial is either explanatory or pragmatic [15], there is a continuum rather than a dichotomy between explanatory and pragmatic trials with the pragmatic attitude explicitly favouring design choices that maximise applicability of the trial's results to usual care settings. As Schwartz and Lellouch wrote:

' [m]ost trials done hitherto have adopted the explanatory approach without question; the pragmatic approach would often have been more justifiable'.

As summarised in [16-18], we are aware of only a single study that has attempted to identify pragmatic trials (identified using: MeSH term 'clinical trial', keyword 'pragmatic' and authors' judgement that identified studies described clinical trials with a pragmatic attitude) and it found just 95 published between 1976 and 2002 [19]. Since PubMed identifies over 168,000 RCTs for that period, trials with a pragmatic attitude are clearly the exception even if we make allowances for Vallvé *et al*.'s rather narrow search [19]. This is at least in part due to the requirements of regulatory agencies, especially the US Food and Drug Administration (FDA) [16-18]. Although the FDA offers little guidance on the design of trials, what guidance there is argues against trials with a pragmatic attitude: ' [T]here are numerous ways of conducting a study that can obscure differences between treatments, such as poor diagnostic criteria, poor methods of measurement, poor compliance, medication errors, or poor training of observers. As a general statement, carelessness of all kinds will tend to obscure differences between treatments. Where the objective of a study is to show a difference, investigators have powerful stimuli toward assuring study excellence' [20]. The FDA equates explanatory design choices with study excellence, thereby favouring trials that lack the attributes needed to support decisions about the applicability of a treatment or therapy to usual practice [16-18]. Conversely, the clinical, policy and funding decision makers who are expected to use these trials for real world funding and clinical decision making are not convinced of their relevance and applicability to their patients or settings [14].

How might a trial with a pragmatic attitude be more helpful to policymakers, clinicians and patients than an explanatory trial? Below we recount two trials demonstrating some of the problems created when trials are not widely applicable.

Consider the Vioxx Gastrointestinal Outcomes Research (VIGOR) trial, which assessed whether rofecoxib (Vioxx) was associated with a lower incidence of upper gastrointestinal events than the non-selective non-steroidal anti-inflammatory drug (NSAID) naproxen among patients with rheumatoid arthritis [21]. The patients with rheumatoid arthritis included in this trial were highly selected; in particular, those with recent cardiovascular events and those taking aspirin were excluded. Patients were followed up for an average of 8 months. Despite VIGOR showing an increased risk of cardiovascular events in patients taking rofecoxib, this was attributed to the protective effect of naproxen. It was not until a later trial of rofecoxib with longer follow-up, the Adenomatous Polyp Prevention on Vioxx (APPROVe) trial, modified its protocol to include patients at higher baseline risk for a cardiovascular event to be enrolled that the increased cardiovascular risk became undeniable and rofecoxib was withdrawn from the market [22]. Had VIGOR taken a more pragmatic approach to participant selection and follow-up, it is likely that the balance of benefit and harm for refecoxib would have been evaluated differently, and far fewer people would have been exposed to these risks.

The National Institute of Neurological Disorders and Stroke (NINDS) trial found a benefit for thrombolytic therapy when used with acute ischemic stroke patients who have had symptoms for less than 3 h [23]. However, in clinical practice, a minority of patients present within 3 h. Moreover, the recruitment protocol of the NINDS trial required 50% of participants to have presented within 1.5 h, a group that is almost non-existent in practice [24]. These design features practically guaranteed that the result of this trial would have poor applicability to the patients more typically seen in clinical practice, necessitating trials with wider inclusion criteria such as International Stroke Trial 3 (IST-3) [25].

The problem of applicability is enlarged when we consider guidelines, where many trials contribute to each recommendation. Travers *et al*. looked at the extent to which community-based asthma and chronic obstructive pulmonary disease (COPD) patients, respectively, would be eligible for the 17 major trials cited in the Global Initiative for Asthma (GINA) guidelines [26] or the 18 major trials cited in the Global Initiative for Chronic Obstructive Lung Disease (GOLD) guidelines [27]. Of the 749 individuals responding to their survey, a median 4% with current asthma (range 0% to 36%) and a median 6% (range 0% to 43%) with current asthma on treatment met the eligibility criteria for the GINA trials. For the GOLD guidelines, a median 5% (range 0% to 20%) with COPD and a median 5% (range 0% to 9%) of those with COPD receiving treatment met inclusion criteria. Such restrictive entry criteria make it very difficult for clinicians to use such guidelines.

Using narrow inclusion criteria for a trial may be appropriate if: (a) there is evidence to support a strong relationship between the selection criteria and treatment response, (b) the criteria used for selection are reasonably common among the typical patient population, both for reasons of trial feasibility and for reasons of population impact, and (c) a typical clinician caring for patients with this condition could easily use these criteria to select patients. In the absence of selection criteria with these traits, the best estimate of treatment effect under real world conditions, is, for any individual patient, the average treatment effect of an intervention on an unselected group of patients with that condition, rather than the treatment effect found in a small and narrow subgroup, defined by multiple exclusion criteria. There are three reasons for this counterintuitive conclusion. Firstly, any single patient to whom we wish to apply the results of a trial is far more likely to be found within the ranks of unselected patients included in a pragmatic trial, than in the highly selected patients of an explanatory trial. Secondly, even though we have substantial epidemiological knowledge about the prognostic factors for disease incidence and outcomes in a population, we have far less knowledge of the clinical and biological characteristics of patients, which determine their treatment response. Thirdly, even in those instances where we know a prognostic factor which influences treatment response, few such factors are overwhelmingly powerful, and sufficiently common to be relevant on a large scale. For those few that are, it is rare to find any which can be implemented in a programmatic fashion once the intervention is proven for the group with that factor.

Our point is not that there should be no explanatory trials. One can argue that most first trials of a healthcare intervention with an obvious and well understood mechanism of action should be small, rapidly conducted pilot trials towards the explanatory end of the explanatory-pragmatic continuum [28]. If this trial rules out a benefit in a select group of patients, treated under ideal conditions, who are thought, based on mechanistic reasoning, to be most likely to benefit then there is no need for more trials. But if the intervention does show a benefit it is still unclear whether it works in the real world, which is why a trial at the pragmatic end of the explanatory-pragmatic continuum is then needed. This trial should involve participants (both clinicians and patients) who are like those for whom the intervention is relevant in the real, messy world of clinical practice [18]. This pragmatic trial should use the current accepted treatment as the comparator, require no more financial or staff resources than are currently available in the type of practice or clinic expected to deliver the new intervention, and the trial should measure an outcome that is of immediate importance to both patients and clinicians [18].

If an intervention is not well understood, or if the intervention has been used in another indication, and the mechanism by which it will provide benefit on a new indication is not clear, then a more pragmatic trial is the place to start. This can be followed, if subgroup analysis reveals startlingly different results in some participant groups, by a trial in which all participants lie within the group who obtained unique benefit.

## Designing pragmatism

There is broad agreement on the type of design decisions that make a trial explanatory or pragmatic in attitude [4,11,14,15,28-32], and Table 1 shows some key differences. Trialists who describe their trials as pragmatic have made design decisions that they believe will make it more likely their trial will achieve its purpose of informing real world decision about which among the alternative treatments to choose [33-37].

**Table 1:** Key differences between trials with explanatory and pragmatic attitudes (from Zwarenstein *et al*. [48]).

	Explanatory attitude	Pragmatic attitude
Question	Efficacy: can the intervention work?	Effectiveness: does the intervention work when used in normal practice?
Setting	Well resourced, 'ideal' setting	Normal practice
Participants	Highly selected; poorly adherent participants and those with conditions which might dilute the effect are often excluded	Little or no selection beyond the clinical indication of interest
Intervention	Strictly enforced and adherence is monitored closely	Applied flexibly as it would be in normal practice
Outcomes	Often short-term surrogates, or process measures	Directly relevant to participants, funders, communities and healthcare practitioners
Relevance to practice	Indirect: little effort is made to match the design of the trial to the decision making needs of those in the usual setting in which the intervention will be implemented	Direct: the trial is designed to meet the needs of those making decisions about treatment options in the setting in which the intervention will be implemented

But how does a trialist with this goal in mind know that his or her trial does indeed have the right design for its purpose? There are at least two tools available to help trialists and others judge where on the explanatory-pragmatic continuum a trial is best placed though they have somewhat different aims. The first, developed by Gartlehner *et al*. [30] characterised trials as efficacy (explanatory) or effectiveness (pragmatic) trials, and was designed to classify trials for systematic review and to help clinicians judge the applicability of trial results. The tool has seven criteria considered relevant to judgements as to where a trial is placed on the efficacy-effectiveness continuum. These include considerations of the trial setting, its inclusion criteria, the choice of health outcome and the length of follow-up. The authors asked the directors of 12 evidence-based practice centres (centres that conduct systematic reviews) in the USA and Canada to nominate 6 trials each: 4 effectiveness studies and 2 efficacy trials. Then, 2 independent raters applied the tool's 7 criteria to the 24 trials that met the study's eligibility criteria. A score of 6 criteria met gave the best balance between sensitivity and specificity for identifying effectiveness trials; at this threshold the tool identified 13 of 18 trials judged to be effectiveness trials by the 12 directors. Used in this dichotomous fashion, however, the tool does tend to reinforce the misconception that a trial is either explanatory or pragmatic, rather than acknowledging that there is a continuum. It has the added problem that one criterion is whether or not the setting of the trial is in primary care, implying that a trial conducted in, say, a referral hospital cannot be oriented towards asking questions of real world effectiveness, even though many patients are treated in such settings.

**Figure 1:**
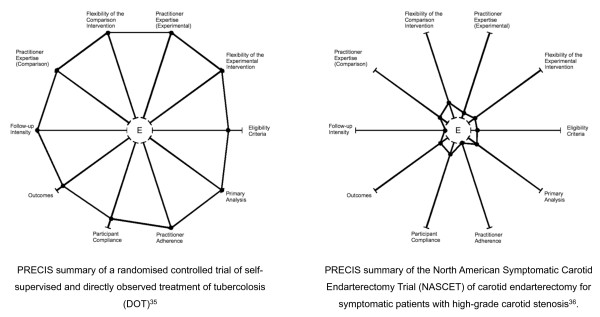
**PRECIS diagrams (based on Thorpe et al **[29]).

A more recent tool, the pragmatic-explanatory continuum indicator summary (PRECIS) [29], (figure 1) is intended to be used by trialists designing a trial to assess the degree to which their design decisions align with the trial's stated purpose. This tool has 10 dimensions based on trial design decisions (for example, participant and practitioner expertise, flexibility with which the intervention can be delivered and choice of comparator), and presents these on a graphical, 10-spoked 'wheel'. A highly pragmatic trial is out at the rim, while explanatory trials are nearer the hub. Table 1 compares the highly pragmatic Directly Observed Treatment (DOT) trial [38] with the highly explanatory North American Symptomatic Carotid Endarterectomy Trial (NASCET) [39]. The advantage of this graph is that it quickly highlights inconsistencies in how the 10 dimensions will be managed in a trial. For example, if the DOT trial had intensely monitored compliance and intervened when it faltered, a single glance at the wheel would have immediately identified this inconsistency with the trial's otherwise pragmatic attitude. This allows trialists to make adjustments, if possible and appropriate, to the design to obtain greater consistency with their trial's purpose.

## Describing context

While these tools do allow a trialist to assess the likely impact of his or her design decisions on the trial's ability to achieve its purpose, they do not address an important observation made by Karanicolas *et al*. that how pragmatic a trial is depends on perspective and context [40]. There is disagreement as to which of perspective and context is the more important [40-43], although both are clearly relevant to someone trying to interpret a trial result. However, while perspective is a feature of the individual reading the trial report and hard for trialists to predict, context (the distinctive features of a trial's setting, participants, clinicians and other staff) is a feature of the trial itself and should be within the capabilities of trialists to describe. We would argue, therefore, that trialists should not worry about trying to guess the various perspectives of those making decisions but should instead do all they can to describe the context of their trial.

Two examples will help to illustrate this point. In a Dutch pragmatic trial comparing web-based self-help for problem drinking with a six-page, web-based psychoeducational brochure on alcohol [44], one of the inclusion criteria for the trial was that participants must have internet access. While this might not be considered a restrictive criterion in the Netherlands, where internet penetration in 2007 was 88% [45], this would not be the case in, say, Poland, where internet penetration is just under 30% [46]. From the perspective of a clinician or policymaker in Poland, one could imagine that a trial conducted in the Dutch context is more explanatory, given the limited penetration of the internet in Poland. Trialists based in the Netherlands cannot be expected to know the perspectives of decision makers in Poland but they can be expected to recognise that an internet penetration of around 88% is part of the context of their trial and report it, which was not performed in this example [44]. Another example is a pragmatic trial performed in Quebec, Canada, which compared a pharmacist-managed anticoagulation service with usual care delivered by general practitioners [47]. Although these trialists found that care was similar for both groups, they also found that pharmacist-managed care was more expensive. However, as the authors report, this is context-specific because the comparator care provided by physicians was performed through telephone consultation, for which physicians receive no monetary compensation in Quebec. Without this contextual information some readers may conclude that the intervention is not applicable to their contexts; with it they may see an opportunity for improving the delivery of care.

Decisions about applicability depend on readers being able to assess the feasibility of the intervention in their own context [48]. However, understanding what comprises the intervention (and often the comparator) is not always a simple matter of reading the trial report [49]. Detailed reporting of the content of interventions, especially complex, non-pharmacological ones, is often poor [49-51]. For example, 41 of 80 published descriptions of studies selected for abstraction by the journal *Evidence-Based Medicine *from October 2005 to October 2006 failed to adequately describe all elements of the intervention [49]. A study of 47 trials involving nurses found that information about the nurses delivering interventions (for example, qualifications, experience, training) was often lacking [52]. This is important contextual information without which it is extremely difficult for readers to make informed judgements about applicability; indeed, it may be impossible.

The recent Consolidated Standards of Reporting Trials (CONSORT) Statement extension for the reporting of pragmatic trials should go some way to improving the reporting of contextual information [48], especially the recommendation for reporting information about the participants and on applicability (or generalisabilty) of the trial findings. Initiatives such as the Workgroup for Intervention Development and Evaluation Research (WIDER) [53], the CONSORT extension for non-pharmacological treatments [51] and the Standards for QUality Improvement Reporting Excellence (SQUIRE) Statement [54] are likely to help others to both judge the applicability of an intervention to their own setting and implement it should they choose to. A trial report with a poor description of the intervention is effectively rendered useless because implementing it elsewhere becomes a matter of guesswork. Readers need to know 'who, what, when and where' [49].

## Assessing applicability

For pragmatic trials, where the intention is to interfere as little as possible with the usual process of care, understanding context is essential. But how are its effects measured? Despite its importance, there is little work exploring how context might influence the results of a trial, or the feasibility of widespread implementation.

The Normalisation Process Model [55-58] may be able to help. The model was developed to guide the design of evaluations of the implementation of complex interventions but applies equally to the study of simple interventions that have complex requirements of the healthcare system needed to deliver them. It may also be adapted to guide the investigation of the feasibility of interventions in advance of their implementation, as it assists in the systematic and comprehensive mapping of the human, organisational and resource changes that an intervention will require. Some interventions can only be implemented with major structural or organisational changes to healthcare delivery; trials evaluating these interventions might be called 'aspirational'. The Normalisation Process Model could help to identify such interventions and allow trialists and others to better judge whether the required changes are feasible on a wide scale and whether the likely benefit of the intervention justifies making them. Documents linked to trial reports could provide empirical data, both quantitative and qualitative, on the features of health care providers, patients or working practices which influenced the observed results, putting judgements about the feasibility of interventions (and hence applicability of the trial's results) on a firmer basis.

The applicability of trial results can also be estimated through statistical modelling. Here the influence of one or more features of a trial, such as the selection of participants, is investigated using statistical techniques to see how sensitive the trial result is to the feature or features being varied. For example, Greenhouse *et al*. have developed techniques for making what they call generalisabilty judgements, which are based on comparisons between RCT participants and individuals included in large surveys, databases and epidemiologic studies that are known to be representative of the population of interest [59]. These authors were interested in a question familiar to users of trials with a pragmatic attitude: how similar are the trial's participants to those of the target population in general? This is clearly relevant to applicability. Greenhouse *et al*. compared the demographic profiles of youths included in trials of antidepressants with the profiles of depressed adolescents contained in a national database, the Youth Risk Behaviour Survey. Although both the trial and survey populations were found to be similar for most demographic characteristics, the rate of suicidal ideation and suicidal behaviours (the trial's primary outcomes) in the trial participants was found to be about half the adjusted rate among depressed adolescents in the USA as estimated from the national database (3.6% vs 7.1%) [59]. This difference appeared to be due to trials excluding adolescents considered to be at high risk of suicide. Although one might reach the same general conclusion of limited applicability after using, say, the PRECIS tool [29], the technique used by Greenhouse *et al*. provides a quantitative estimate of applicability, at least with regard to participant selection. Other aspects of applicability have also been considered using quantitative methods. Yamaguchi and Ohashi used a proportional hazards model to investigate the influence of treatment-by-centre and baseline risk on the trial result in a multicentre superficial bladder cancer trial [60]. Yamaguchi and Ohashi found that although there was some variation between centres, especially in the baseline risk, this made little difference to the estimate of treatment effect. While we shouldn't overstate the predictive power of modelling on the benefits of an intervention applied outside the trial's original context, it does have a role to play.

## Conclusion

An internally valid trial that has poor applicability, or is reported in such a way that it is difficult or impossible for others to make judgements about its applicability, is a lost opportunity to influence clinical practice and healthcare delivery. It is worth repeating a line from Rothwell's 2005 *Lancet *paper: 'Lack of consideration of external validity is the most frequent criticism by clinicians of RCTs, systematic reviews, and guidelines' [8]. An increase in the number of well-designed trials with a pragmatic attitude is surely needed. Perhaps the FDA and other regulatory authorities might also consider revisiting the gap in their regulations on the design of trials whose goal is to support decision making. The FDA's dismissal of much of the reality of real-life clinical practice as carelessness to be avoided in a trial does not help a trialist who wants to design a trial that can be used by policymakers and clinicians to decide which of several competing treatments they should be using in the unkempt world of usual practice.

Some trials aim to provide data on whether an intervention can be effective under optimal conditions; these trials have an explanatory attitude. Others aim to show that an intervention is effective in real and far from ideal conditions; these trials have a pragmatic attitude. Both attitudes have their place. However, we believe that:

1. More trials should have a pragmatic attitude.

2. Trialists should give as much care and attention to issues of applicability as they already do to issues of internal validity.

So, what should trialists do to improve the applicability of their trials? Trialists should routinely ask themselves at the design stage of their trial 'Who are the people I expect to use the results of my trial and what can I do to make sure that these people will not be forced to dismiss my trial as irrelevant to them, their patients, or their healthcare systems?' Rothwell gives a good list of issues that affect applicability [8], and Table 1's 'Pragmatic attitude' column gives pointers to design issue that can increase applicability, as does the CONSORT extension for pragmatic trials [48]. The PRECIS tool [29] and that by Gertlehner *et al*. [30] can help trialists to match design to purpose. While there is some evidence suggesting factors that have influenced applicability, there is not enough empirical study of this question, and we are in need of a body of work similar to that performed over the past decades on internal validity. We would suggest that attention is given to the following:

1. Summarising existing evidence on the relevance of trials to decision making within a trial's own context and, if available, on relevance to other contexts.

2. Developing a methodology for identifying contextual factors of importance and estimating their influence on applicability.

3. Developing standards for describing and reporting contextual information.

Wells provides a list of research recommendations linked to trials of complex interventions, which is also relevant [61].

Users of trial reports need to make judgements about the applicability of the results to their own context, a task to which those designing the trial often give insufficient thought. If we want evidence from trials to be used in clinical practice (and we do), trialists should make every effort to make their trial widely applicable, which means that more trials should be pragmatic in attitude [16-18]. Trialists should not give policymakers, clinicians and patients reason to ignore research evidence.

## Competing interests

The authors declare that they have no competing interests.

## Authors' contributions

Both authors contributed to the design of the paper. ST wrote the first draft. Both authors critically reviewed and edited drafts and read and approved the final manuscript.
